# Adult *Hymenolepis nana* and its excretory–secretory products elicit mouse immune responses via tuft/IL-13 and FOXM1 signaling pathways

**DOI:** 10.1186/s13071-025-06719-w

**Published:** 2025-03-11

**Authors:** Rong Mou, Xuan-Yin Cui, Yu-Si Luo, Yi Cheng, Qing-Yuan Luo, Zhen-Fen Zhang, Wen-Lan Wu, Jin-Fu Li, Ke Zhang

**Affiliations:** 1 The Guizhou Key Laboratory of Microbio and Infectious Disease Prevention & Control/The Key and Characteristic Laboratory of Modern Pathogenicity Biology, Departments of Parasitology & Histology and Embryology, School of Basic Medical Sciences, Guizhou Medical University, Room 220, E-1 Building, Ankang Avenue No. 6, Guiyang, 561113 China; 2 Emergency ICU, The Affiliated Hospital of Guizhou Medical University, Guiyang, 550004 China; 3 Department of Emergency, Liupanshui Hospital of the Affiliated Hospital of Guizhou Medical University, Liupanshui, 553000 China; 4 Center for Tissue Engineering and Stem Cell Research, Guizhou Medical University, Guiyang, 561113 China

**Keywords:** *Hymenolepis nana*, Immune responses, Excretory-secretory products, RCM-1, Tuft/IL-13, FOXM1

## Abstract

**Background:**

Hosts typically elicit diverse immune responses to the infection of various parasitic worms, with intestinal epithelial cells playing pivotal roles in detecting parasite invasion. *Hymenolepis nana* (*H*. *nana*) is a zoonotic parasitic worm that resides in the host’s intestine. The contribution and underlying mechanisms of tuft cell-mediated immune reactions against *H*. *nana* remain unexplored.

**Methods:**

This study endeavors to examine the immune responses in the mouse intestine elicited by the adult *H*. *nana* and its excretory–secretory products (ESP). Ileal tissue alteration was detected using hematoxylin and eosin (H&E) staining, changes in the number of intestinal stem cells, goblet cells, tuft cells, and Paneth cells were detected by immunohistochemistry (IHC), immunofluorescence (IF), etc., and changes in the expression of type 2 cytokines and FOXM1 were detected by Western blotting (WB) or real-time quantitative polymerase chain reaction (RT-qPCR).

**Results:**

The presence of adult *H*. *nana* and its ESP enhanced the number of tuft cells and goblet cells while fostering the production of type 2 cytokines. Furthermore, the surge in Paneth cells and FOXM1 triggered by *H. nana* aids in maintaining intestinal stem cells homeostasis and proliferation. Notably, the FOXM1 inhibitor RCM-1 dampened intestinal stem cells differentiation and type 2 cytokines secretion, potentially impeding the host's capacity to eliminate *H*. *nana*.

**Conclusions:**

The adult *H*. *nana* and its ESP stimulate the immune responses in mice through tuft/interleukin (IL)-13 and FOXM1 signaling pathways and promote the elimination of *H*. *nana* from the host through the differentiation of intestinal stem cells into tuft cells, goblet cells, and Paneth cells, as well as the activation of type 2 immune responses. Meanwhile, RCM-1 inhibits the immune responses to *H*. *nana* in mice, thus affecting the excretion of *H*. *nana* by host.

**Graphical Abstract:**

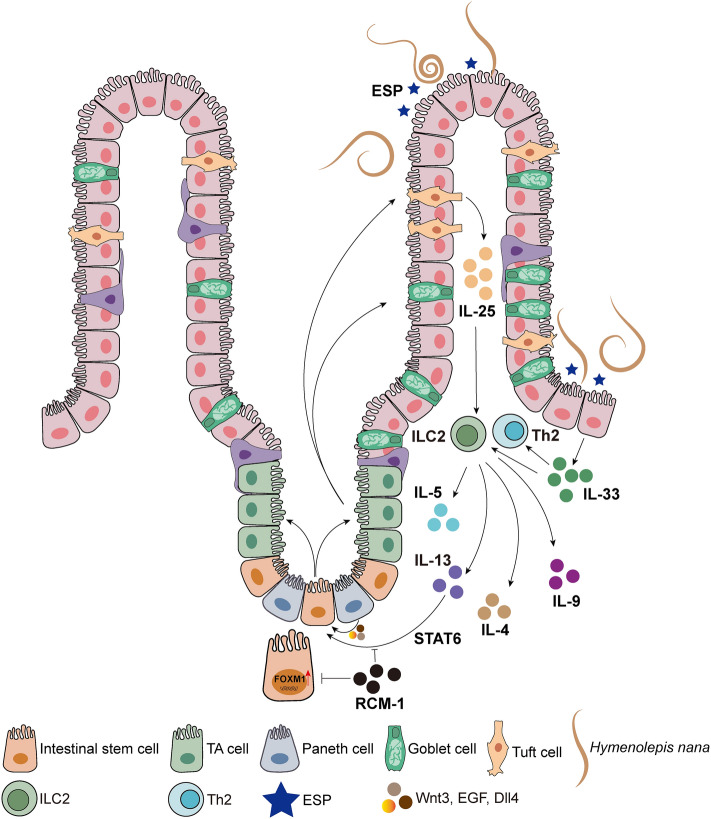

**Supplementary Information:**

The online version contains supplementary material available at 10.1186/s13071-025-06719-w.

## Background

Parasitic worms are among the most common pathogens in nature. To complete their life cycle, these intestinal worms traverse host tissues, causing severe damage, which imposes a significant burden on global health systems [1]. The host’s resistance to intestinal pathogens relies on the immunoregulatory functions of immune cells. Innate lymphoid cells (ILC) are tissue-resident immune cells that are early responders to infection. The classic ILC subtypes are divided into three groups: group 1 ILC (ILC1) predominantly secretes interferon gamma (IFN)-γ, group 2 ILC (ILC2) predominantly secretes interleukin (IL)-13, and group 3 ILC (ILC3) predominantly secretes IL-22, and which host ILC predominates depends on the type of intestinal pathogen the host is infected with [2]. Tissue damage caused by intestinal worm infections triggers epithelial cells to produce alarmin cytokines IL-25 and IL-33, which in turn activate ILC2 [3]. The cytokines secreted by ILC2 or Th2 cells can also directly influence intestinal epithelial cells and various immune cells to drive specific immune responses [4]. The intestinal epithelium consists of various cell types responsible for nutrient absorption and providing a protective barrier, as well as being able to rapidly change its cellular composition to defend against pathogen invasion. Intestinal epithelial cells undergo rapid renewal, with a turnover every 3–5 days [5]. Intestinal stem cells (ISC) are primarily located in the intestinal crypts, and play a crucial role in the renewal process of intestinal epithelial cells, differentiating into all intestinal epithelial cell types, including enterocytes, enteroendocrine cells, tuft cells, goblet cells, and Paneth cells [6, 7]. Paneth cells are the only differentiated cells in the crypts and are interspersed with the ISC, and secrete epidermal growth factor (EGF), Wnt3, and the Notch ligand Dll4 to maintain ISC homeostasis [8].

Intestinal tuft cells are chemosensory epithelial cells that have garnered significant attention in the study of host–parasite interactions. Tuft cells are crucial for defending worms, as their numbers increase sharply during intestinal parasite infections. They are also the primary source of intestinal IL-25, which plays a role in controlling the number of ILC2 during worm infection [9]. ILC2 can secrete type 2 cytokines, including IL-4, IL-5, IL-9, and IL-13 [10]. Tuft cells serve as vital sentinels in the gastrointestinal tract, rapidly proliferating after exposure to type 2 cytokines and playing a key role in protecting against worm infections [11]. This ultimately forms a positive feed-forward loop, where IL-25 produced by tuft cells activates ILC2, and IL-13 produced by ILC2 induces the differentiation of ISC into tuft cells [12]. RCM-1 is an inhibitor of the IL-13/STAT6 signaling pathway [13], and IL-13 induces proliferation and differentiation of ISC through downstream STAT6 signaling [14]. RCM-1 is also an inhibitor of FOXM1, a positive regulator of cell proliferation in various tissues including the intestinal epithelium [13, 15].

In response to intestinal parasites residing in the small intestine, IL-13 produced by immune cells increases and induces ISC to differentiate into tuft cells and goblet cells [16]. Goblet cells are key components of the host’s defense against parasites, producing and releasing mucins that form a dense mucus layer on the surface of the intestinal mucosa, working alongside other cells to maintain intestinal homeostasis. After worm infection, the proliferation timelines of tuft cells and goblet cells are synchronized [17]. The damage caused by helminths to the host is primarily divided into mechanical damage induced by the helminths and the effects of a series of immunomodulatory molecules secreted by the helminths, collectively referred to as excretory-secretory products (ESP) [18].

*Hymenolepis nana* (*H*. *nana*) is a zoonotic parasite that parasitizes the intestines of humans and rodents, causing hymenolepiasis. Mild infections of *H*. *nana* in humans have no obvious clinical symptoms, while severe infections manifest as abdominal pain, diarrhea, anemia, and fever [19, 20]. *Hymenolepis nana* infects people of all age groups, with a predominance of infections in children under 10 years old [21]. It is estimated that the number of infected people worldwide is estimated to be 50–75 million and is more pronounced in Asia, Africa, Southern/Eastern Europe, and Central/South America [22]. After infection with *H*. *nana*, the oncosphere in the eggs invades the intestinal villi, develops into cysticercoid on day 4 of infection, and enters the intestinal lumen, where they mature on about day 12 [23]. It is unclear how *H*. *nana* relates to the host immune responses and whether the immune responses to *H*. *nana* in mice will be suppressed with the RCM-1.

In the current study, we investigated the effects of *H*. *nana* adult worm infection and intraperitoneal injection of adult *H*. *nana*-derived ESP on the host. We found that both the adult worms and ESP of* H*. *nana* could promote the number of tuft cells, goblet cells, and Paneth cells and activate the type 2 immune responses through the tuft/IL-13 and FOXM1 signaling pathways, whereas RCM-1 suppressed these immune responses.

## Methods

### Reagents

Detailed information on all reagents and antibody sources can be found in reagents in the Supplementary Materials (Additional file 1).

### Animal experiments

All animal experiments of the current study were approved by the Animal Ethics Committee of Guizhou Medical University (approval nos. 2100346 & 2100347).

#### Acquisition of *H*. *nana* from hamsters, and the extraction of adult* H*. *nana*-derived excretory-secretory products (ESP)

The 4–6-week-old male hamsters (*n* = 30) were purchased from one pet market in Nanming District, Guiyang, China, in March 2024. Hamsters were sacrificed under anesthesia to obtain intestinal parasites (all hamsters were infected with intestinal parasites), and the parasites were Identified as *H. nana.* The *H*. *nana*, retrieved from hamsters, underwent rigorous cleaning procedures involving multiple rinses with sterilized PBS. Subsequently, 20–30 adult worms were immersed in RPMI 1640 medium (Yeasen, China), supplemented with 1% penicillin/streptomycin/amphotericin B. The medium was collected after 36 h of incubation at 37 °C in a constant temperature incubator. To concentrate the ESP-rich medium, centrifugation at 4000 × g was executed utilizing a 10 kDa ultrafiltration tube (Millipore, Billerica, MA, USA), with the solvent subsequently exchanged for PBS. Sterility was ensured by passing the ESP solution through a 0.22 μm filter, and the protein content was quantified using a BCA protein assay (Yeasen, China). The prepared ESP was then stored at −80 °C for future applications.

The methodology for the identification of *H*. *nana* and ESP is presented in Additional file 1, and the results are presented in Additional file 3: Figure S2.

#### The C57BL/6J mice experiments

The C57BL/6J mice (specific pathogen free, female, 6–8 weeks old, weighing approximately 22.0 ± 2.0 g) used in this study were obtained from the Experimental Animal Center of Guizhou Medical University [SCXK (Jing) 2019-0010]. These mice were housed in a standard laboratory environment, devoid of parasitic contamination, with a regulated temperature range of 20–22 °C and a controlled 12-h light/dark cycle. After a 7-day period of acclimatization, the experiments commenced.

RCM-1 is an inhibitor of FOXM1 and also blocks IL-13/STAT6 signaling, and its administration and dosing refer to previous literature [13, 24]. The mice were randomly allocated into five groups (*n* = 8/group): control (Ctrl), ESP, ESP + RCM-1, *H*. *nana*, and *H*. *nana* + RCM-1. All mice received sterilized H_2_O for 14 days. Starting from the first day, the ESP + RCM-1 and *H. nana* + RCM-1 groups received daily intraperitoneal injections of 1.7 mg/kg RCM-1 (Selleck, China) for seven consecutive days. Our preliminary experiments indicated that 50 μg/day of ESP was more effective than 25 μg/day (Additional file 2: Figure S1), hence we selected the dose of 50 μg/day for the ESP and ESP + RCM-1 groups, which were administered with intraperitoneal injections of ESP from day 7 onward for 7 days. On the basis of preliminary findings, the *H*. *nana* and *H*. *nana* + RCM-1 groups were orally inoculated with 2000 eggs per mouse on the first day, as this dose resulted in the optimal infection rate. All mice were euthanized on day 14.

### Ileum hematoxylin and eosin (H&E) staining and alien-blue and periodic acid-Schiff (AB-PAS) staining

The ileum tissues of the mice were collected and fixed in 4% paraformaldehyde, embedded in paraffin, and cut into 4 µm thickness sections. According to the reagent instructions to stain, H&E staining (Solarbio, China) was used to observe changes in the small intestinal epithelial villi and AB-PAS staining (Saint-Bio, China) for ileum goblet cells (specific steps are in Additional file 1).

### Real-time quantitative polymerase chain reaction (RT-qPCR)

Total RNA was extracted from mouse ileum tissues using the Trizol protocol (specific steps are in Additional file 1). Subsequently, the total RNA was reversely transcribed into cDNA, and the amplification was detected using the SYBR RT-qPCR Kit (Yeasen, China) in a real-time fluorescence quantitative PCR instrument (CFX96, Bio-Rad, Hercules, CA, USA). The amplification protocol consisted of an initial denaturation step at 95 °C for 5 min, followed by 39 cycles of denaturation at 95 °C for 10 s, and annealing at 60 °C for 30 s. The relative mRNA transcription levels of *Muc2*, *Dclk1*, *IL-25*, *IL-33*, *IL-4*, *IL-5*, *IL-9*, *IL-13*, *Olfm4*, *Lgr5*, *Lyz1*, *Wnt3*, *EGF*, and *Dll4* were semi-quantified by the 2^−ΔΔCt^ method, and *GAPDH* was used as the internal control for normalization. The primer sequences are listed in Additional file 5: Table S1.

### Immunohistochemistry (IHC) and immunofluorescence (IF)

The paraffin-embedded sections of mouse ileum tissues were first deparaffinized using xylene and graded concentrated ethanol. Antigen retrieval was then performed by boiling the sections in 1 × EDTA antigen retrieval solution for 20 min. Following this, the sections were treated with 3% endogenous peroxidase enzyme for 10 min to block endogenous peroxidase activity. For IHC, the sections were blocked with 5% goat serum for 30 min before incubation overnight with primary antibodies specific for MUC2 (Abcam, 1:2000), DCLK1 (Abcam, 1:200), IL-13 (Boster, 1:200), OLFM4 (Cell Signaling Technology, 1:200), GATA3 (HuaBio, 1:200), and lysozyme (LYZ) (Abcam, 1:2000). On the next day, the sections were incubated with the appropriate secondary antibody (MCE, 1:500), followed by DAB staining (zsbio, China). Finally, the sections were mounted with neutral gum and observed using a slide scanner (Olympus SLIDEVIEW VS200, Japan). For IF, the sections were permeabilized with 0.3% Triton X-100 for 30 min and blocked with 5% BSA for 1 h. They were then incubated overnight with primary antibodies with respective specificity for LGR5 (Affinity, 1:100), OLFM4 (Cell Signaling Technology, 1:200), MUC2 (Abcam, 1:500), DCLK1 (Abcam, 1:200), and lysozyme (LYZ) (Abcam, 1:250). The next day, the sections were incubated with fluorescent secondary antibody 488 (Thermo Fisher Scientific, 1:200) and DAPI solution (Solarbio, China). Finally, they were mounted with an anti-fade mounting medium and observed using an upright fluorescence microscope (Eclipse 80i, Nikon Ltd, Japan).

### Western blotting (WB)

The mouse ileum tissues were digested with RIPA lysis buffer. After measuring the concentration, an equal amount of protein samples was loaded and isolated using SDS-PAGE. Next, the proteins were transferred onto a PVDF membrane. After blocking with 5% non-fat milk, the membrane was incubated with the primary antibodies for IL-4 (Proteintech, 1:1000), FOXM1 (Proteintech, 1:3000), and LGR5 (ZenBio, 1:1000) at 4 °C overnight. Then it was incubated with the secondary antibody (MCE, 1:10000) for 1 h at room temperature. Finally, Super ECL Detection Reagent (Yeasen, China) was utilized for sensitive detection of the protein and observed using a Chemiluminescence imager (Bio-Rad, Hercules, CA, USA).

### Statistical analysis

The statistical analysis and graphical representation were performed using GraphPad Prism (Version 5.0). The data were presented as mean + SD. Semi-quantitative statistics used Image J software (version 1.53i, US National Institutes of Health, USA) after image acquisition (specific steps are in Additional file 1). Measurements were first subjected to normality tests, and the homogeneity of variance test was performed between groups. Mann–Whitney *U*-test was employed as a non-parametric statistical method to analyze data that deviated from normal distribution. One-way analysis of variance (ANOVA) was used to test differences between multiple groups and Mann–Whitney *U*-test and *t*-test were used to compare differences between two groups. *P*-values less than 0.05 were considered a statistically significant difference.

## Results

### Experimental design

**Fig. 1:**
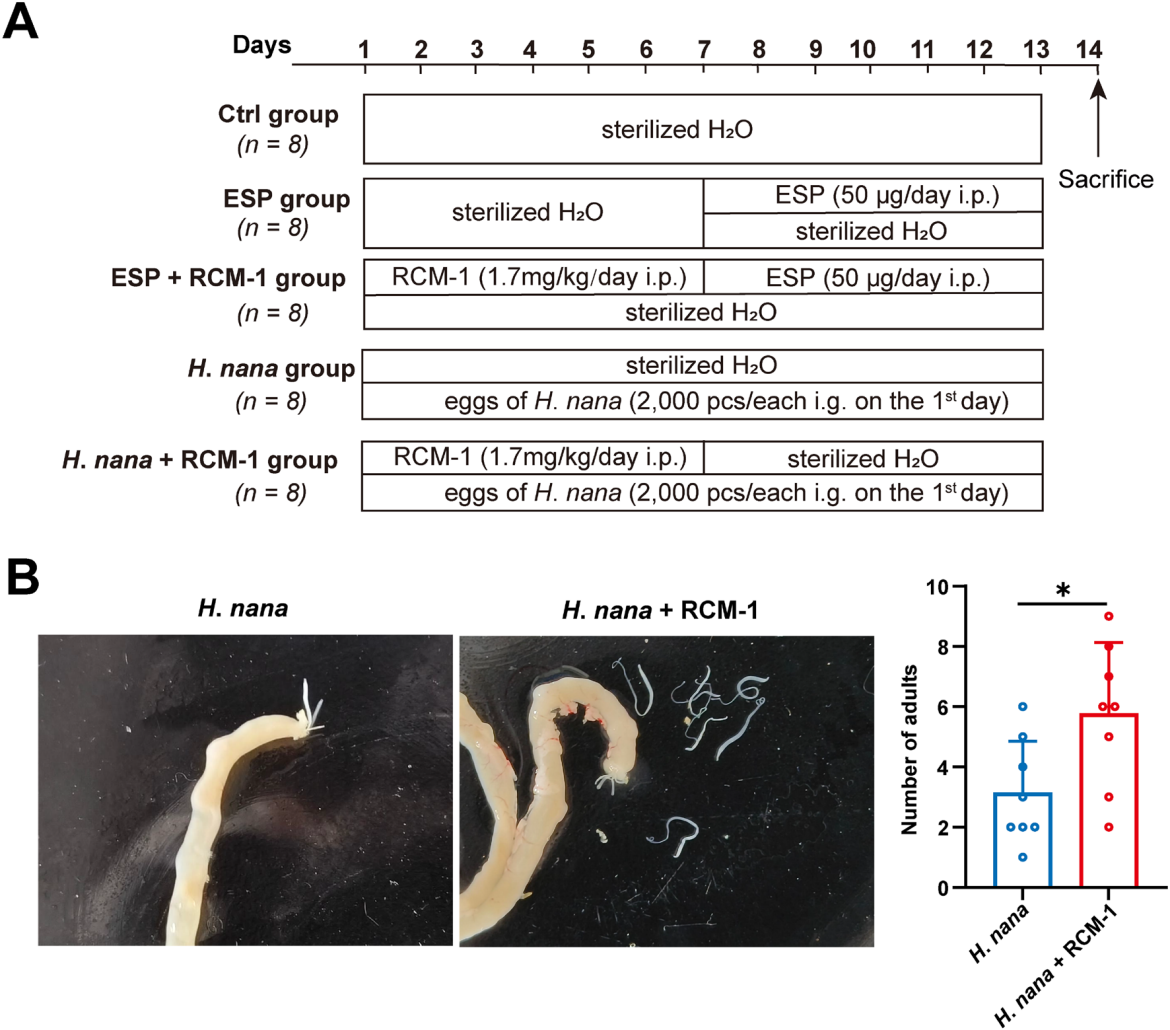
The experimental flowchart and RCM-1 increase the *H*. *nana* infection load. **A** Schematic diagram of experimental design. i.p., intraperitoneal injection; i.g., intragastric injection. **B** The *H*. *nana* adults dissected from mice of *H*. *nana* and *H*. *nana* + RCM-1 groups were shown by pictures (left panel) and the number of *H*. *nana* adults was shown by statistical bar chart (right panel). For the right panel of (**B**), data are presented as mean + SD (*n* = 8 per group), *P < 0.05

Through intraperitoneal injection of ESP in mice, it was found that a dose of 50 μg/day increased the number of goblet cells and tuft cells in the small intestines of mice more than a dose of 25 μg/day (Additional file 2: Figure S1). Therefore, this dosage was selected for subsequent experiments to study the interaction between ESP and the host. To investigate the impact of *H*. *nana* on the intestinal immune responses in mice, the following experimental approach was adopted (Fig. 1A). Successful infection with *H*. *nana* was determined by detecting eggs in fecal samples and dissecting adult worms from the intestinal lumen (Additional file 3: Fig. S2G,H). We found a 100% infection rate for *H*. *nana*, and RCM-1 administration increased the infection load of *H*. *nana* (Fig. 1B).

### RCM-1 restores the shortage of intestinal villi induced by ESP and exacerbates the shortage of intestinal villi induced by adult *H*. *nana*

**Fig. 2:**
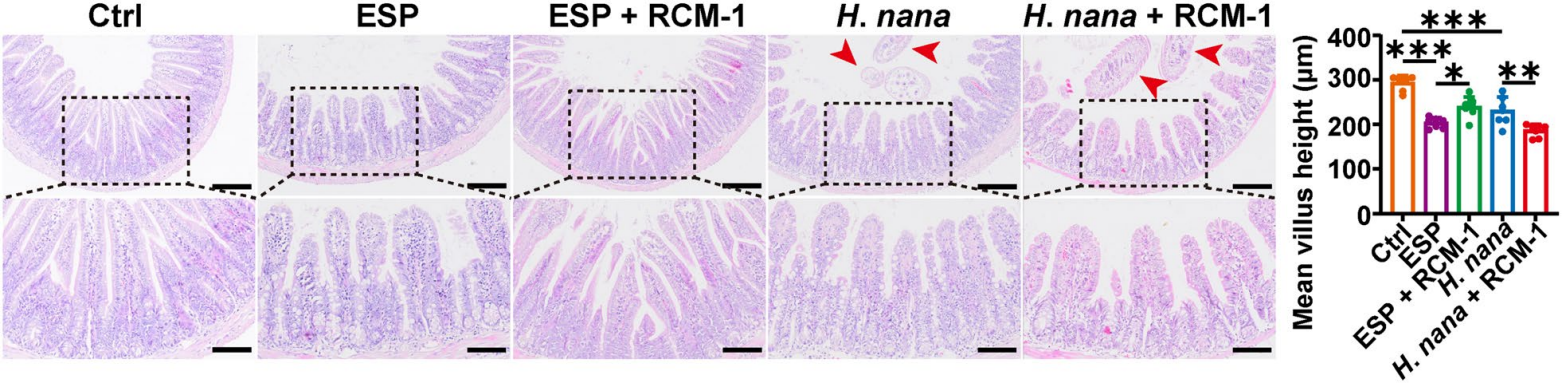
RCM-1 partially restores ESP-induced shortening of the intestinal villi, whereas RCM-1 exacerbates shortening of the intestinal villi induced by *H*. *nana* adult. Representative images of the H&E-stained small intestine (*H*. *nana* is highlighted by red arrowheads, scale bars 200 μm for the upper panel and 100 μm for the lower panel), and mean villus height (μm) were semi-quantified using Image J software. Data are presented as mean + SD for the right panel, *n* = 7 per group, *P < 0.05, **P < 0.01, ***P < 0.001

Intestinal parasitic infections can cause atrophy of the small intestinal villi. To investigate the effects of *H*. *nana* infection and ESP on mouse gut, we used H&E staining to observe changes in the small intestinal villi. We found that *H*. *nana* infection resulted in the presence of adult worm segments in the intestinal lumen. Both *H*. *nana* infection and intraperitoneal injection of ESP led to the shortening of intestinal villi. However, following RCM-1 intervention, the shortening of villi induced by ESP showed partial recovery, while the shortening induced by *H*. *nana* infection was exacerbated (Fig. 2).

### RCM-1 inhibits adult *H*. *nana* and its ESP-induced promotion of goblet cell proliferation and increases MUC2 production

**Fig. 3:**
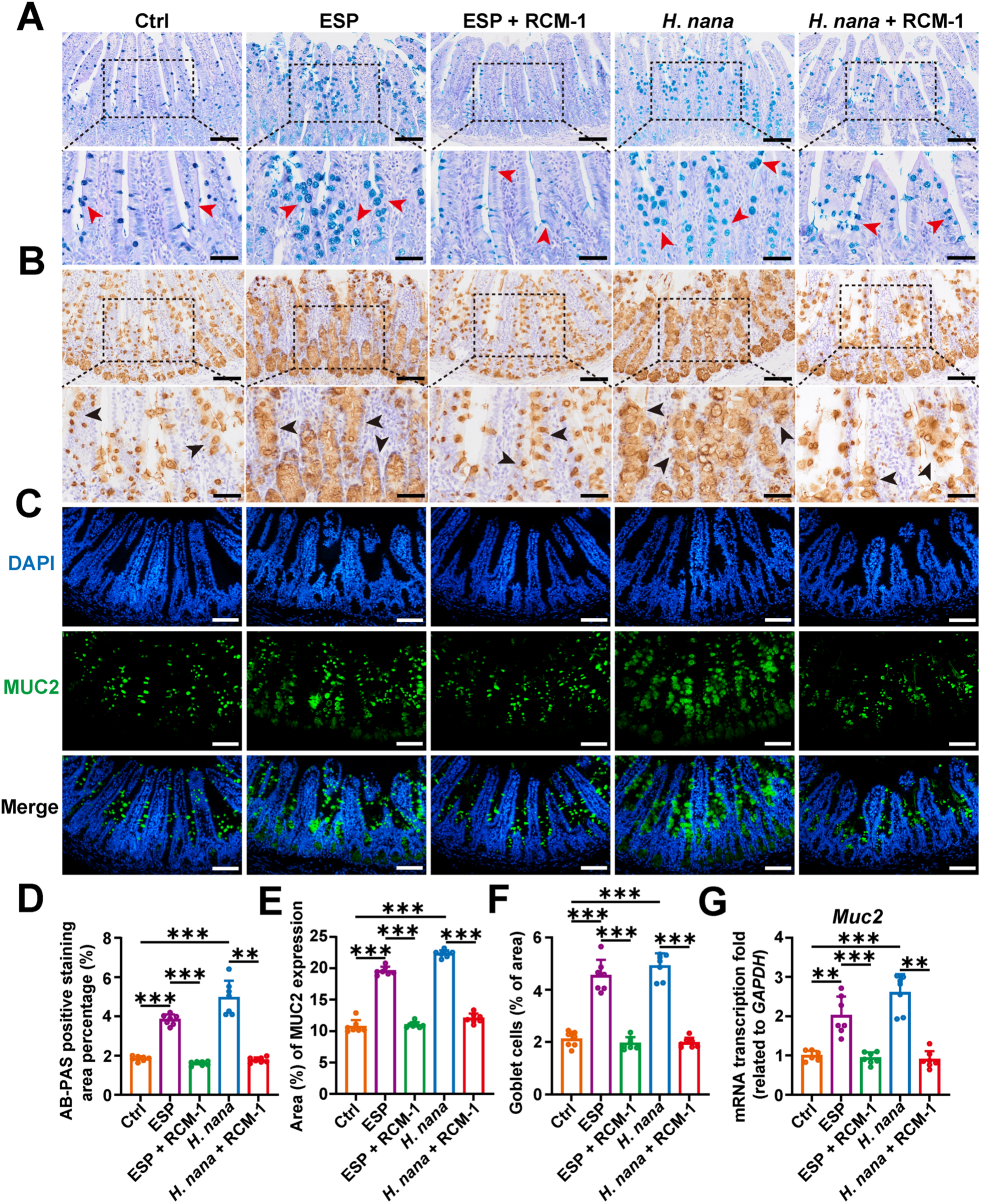
RCM-1 inhibits the promotion of goblet cell hyperplasia and increases MUC2 production induced by the adult *H*. *nana* and its ESP. **A** Representative images of AB-PAS-stained goblet cells (sharp or deep blue highlighted by red arrowheads, scale bars 100 μm for the upper panel and 50 μm for the lower panel). **B** Representative images of IHC with MUC2 (brown highlighted by black arrowheads, scale bars 100 μm for the upper panel and 50 μm for the lower panel). **C** Representative images of IF with MUC2 (green) and the nucleus (DAPI, blue) (scale bars 100 μm). Percentages of the statistics of AB-PAS-stained positive area (**D**), the MUC2-positive area (**E**), and the number of goblet cells (**F**) were semi-quantified using Image J software. **G** The transcription level of *Muc2* and the relative quantification were determined using the 2^−ΔΔCt^ method normalized to *GAPDH*. Data are presented as mean + SD for (**D**–**G**), *n* = 7 per group, **P < 0.01, ***P < 0.001

Goblet cells produce a number of effector molecules including a range of mucins and antimicrobial proteins, which enable these to play a key part in innate defense mechanisms in the gut, against both bacterial and helminth infections [25]. The effect of *H*. *nana* on mouse intestinal goblet cells was investigated using AB-PAS staining, as well as IF, IHC, and RT-qPCR methods to detect the goblet cell marker (MUC2). AB-PAS staining revealed goblet cells in the small intestinal villi stained blue–purple. The results showed that *H*. *nana* promoted an increase in the number of goblet cells in the intestine. However, after the RCM-1 intervention, the number of goblet cells decreased (Fig. 3A and D). This finding was corroborated by IF and RT-qPCR results for MUC2 (Fig. 3C, F and G). The mucin MUC2 secreted by goblet cells was detected by IHC and it was found that RCM-1 inhibited the increase in MUC2 induced by adult *H. nana* and its ESP (Fig. 3B,E). These results suggest that adult *H*. *nana* and its ESP promote goblet cell hyperplasia and increase MUC2 production, while RCM-1 inhibits these changes.

### RCM-1 prevents the increase of mouse small intestinal tuft cells and the cytokines of IL-25 and IL-33

**Fig. 4:**
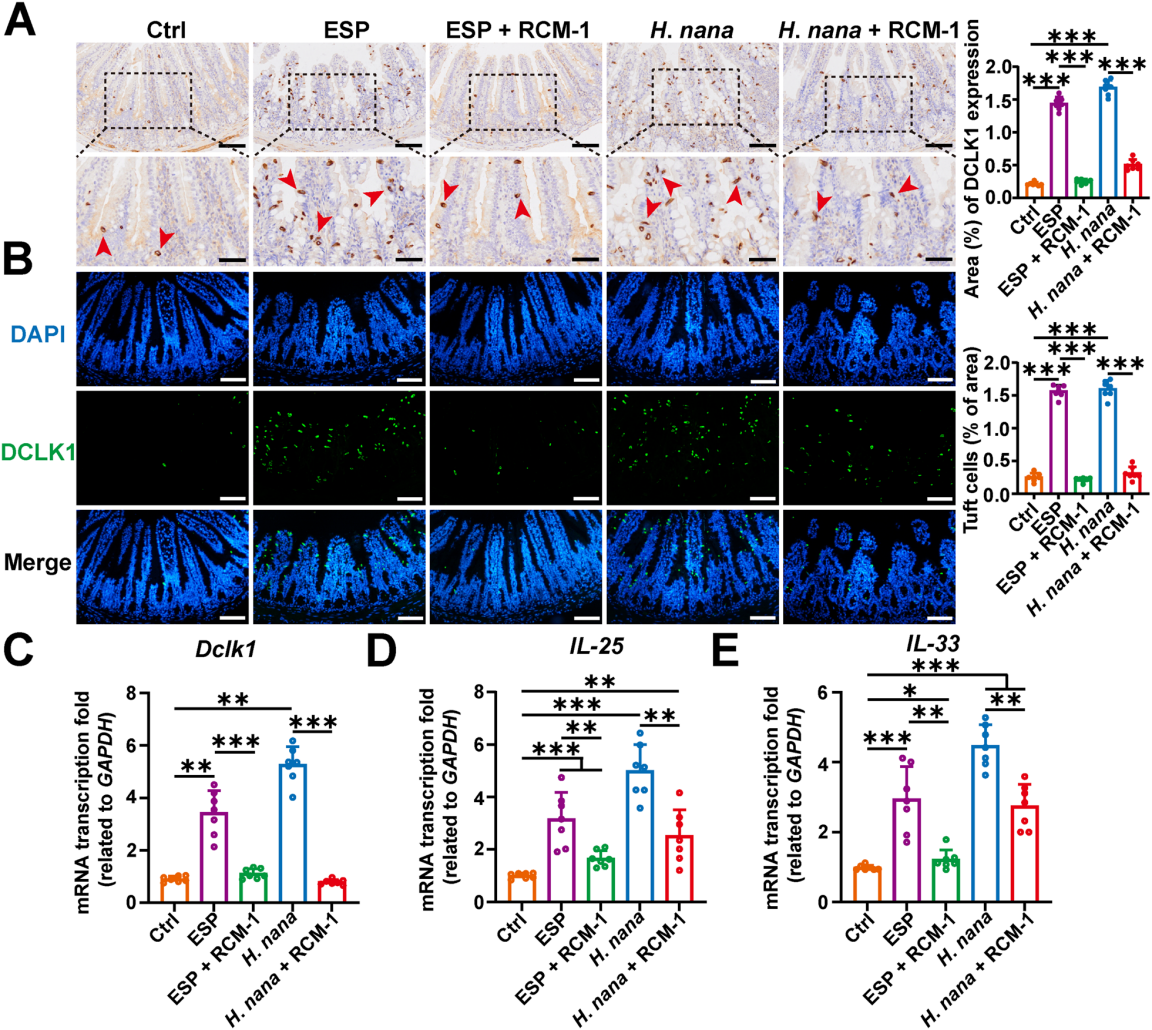
RCM-1 inhibits the increase in the number of small intestinal tuft cells and the transcription levels of *Dclk1*, *IL-25*, and *IL-33* induced by adult *H*. *nana* and its ESP in mice. **A** Representative images of IHC with DCLK1 (brown highlighted by red arrowheads, scale bars 100 μm for the upper panel, and 50 μm for the lower panel). **B** Representative images of IF with DCLK1 (green) and the nucleus (DAPI, blue) (scale bars 100 μm). Percentages of DCLK1-positive area and the number of tuft cells were semi-quantified using Image J software. The transcription levels of *Dclk1* (**C**); *IL-25* (**D**); and *IL-33* (**E**) were determined using the 2^−ΔΔCt^ method normalized to *GAPDH*. Data are presented as mean + SD for (**C**–**E**) and the right panels of (**A**, **B**), *n* = 7 per group, *P < 0.05, **P < 0.01, ***P < 0.001

Recent studies have found that tuft cells play a first-line defense role in certain intestinal parasitic infections [26], but research on tuft cells in *H*. *nana* infections is still lacking. IHC and IF were assayed for tuft cell marker DCLK1, and the results showed that both *H*. *nana* adults and ESP significantly increased the number of tuft cells in the intestine, while RCM-1 inhibited this increase remarkably (Fig. 4A and B). These findings were confirmed by the RT-qPCR approach (Fig. 4C). The cytokines IL-25 secreted by tuft cells and IL-33 secreted by intestinal epithelial cells contribute to the secretion of type 2 cytokines. The results showed that *H*. *nana* infection and ESP both promoted an increase in the transcription levels of *IL-25* and *IL-33* (Fig. 4D,E). These findings suggest that *H*. *nana* increases the number of tuft cells in the mouse intestine along with the expression of alarmin cytokines IL-25 and IL-33, while it can be suppressed by RCM-1.

### *Hymenolepis nana* induces the expression of type 2 cytokines in the mouse small intestine, which is suppressed by RCM-1

**Fig. 5:**
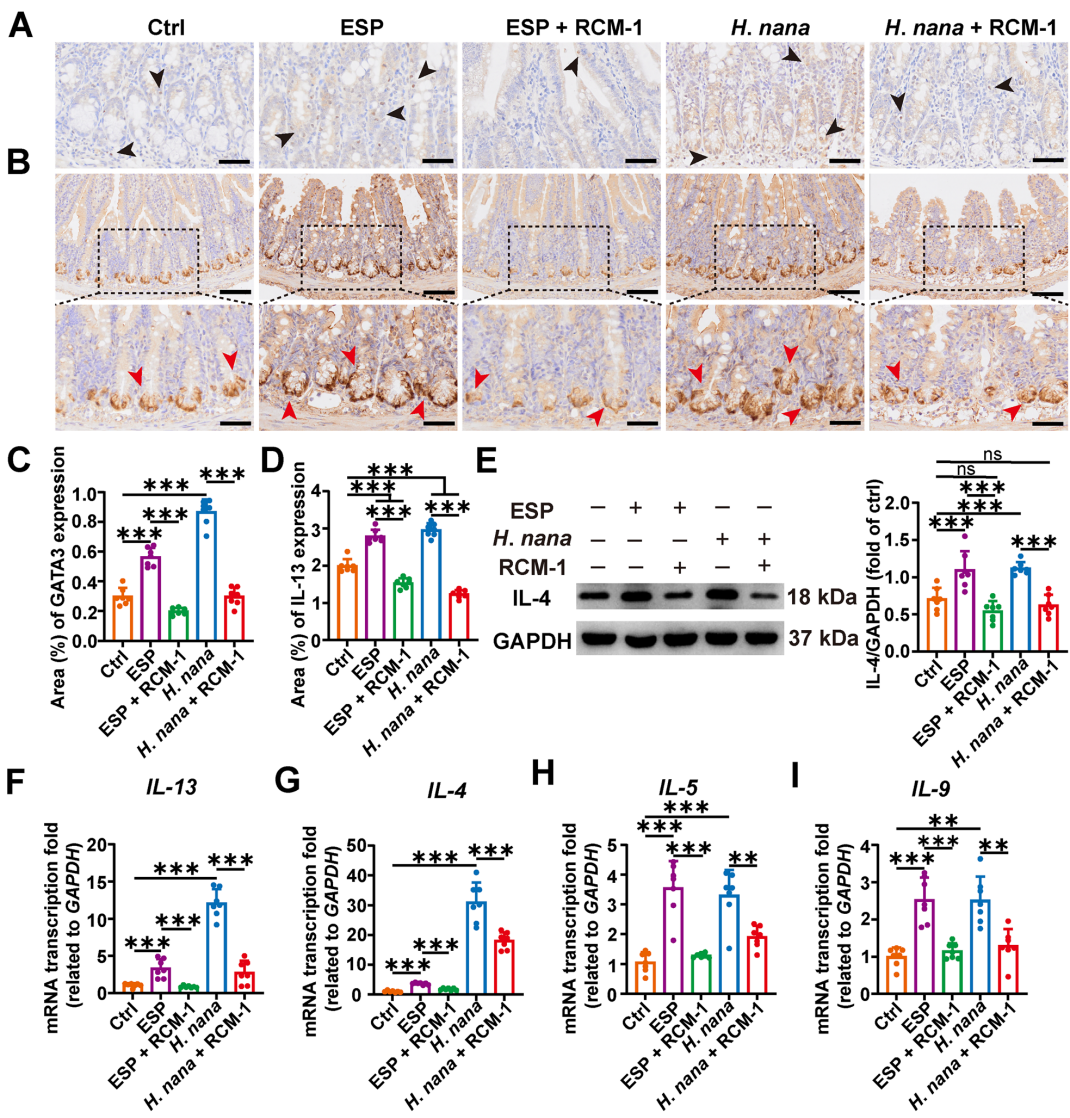
RCM-1 prevents type 2 cytokines expression in mice to expel *H*. *nana*. **A** Representative images of IHC with GATA3 (brown highlighted by black arrowheads, scale bars 50 μm). **B** Representative images of IHC with IL-13 (brown highlighted by red arrowheads, scale bars 100 μm for the upper panel and 50 μm for the lower panel). Percentages of positive-area of GATA3 (**C**) and IL-13 (**D**) were semi-quantified using Image J software. **E** The protein level of IL-4 (left panel) and the percentage of the relative expression of IL-4 were semi-quantified using Image J software (right panel). The relative quantifications of transcription levels of *IL-13* (**F**), *IL-4* (**G**), *IL-5* (**H**), and *IL-9* (**I**) were determined by the 2^−ΔΔCt^ method normalized to *GAPDH*. Data are presented as mean + SD for (**C**–**I**) and the right panel of (**E**), *n* = 7 per group, **P < 0.01, ***P < 0.001; ns, not statistically significant

Type 2 immune responses are essential for protection against intestinal helminth infections, and type 2 cytokines are secreted by either ILC2 or Th2 cells, with insufficient secretion compromising parasite clearance. STAT6-mediated signaling is activated by both IL-4 and IL-13 via the IL-4 receptor and leads to a range of effector mechanisms that promote the expulsion of gastrointestinal helminths [27]. Since RCM-1 acts downstream of the IL-13/STAT6 pathway [13], it can then be speculated that RCM-1 also interferes with the IL-4-activated STAT6 pathway. The transcription factor GATA3 is upregulated in ILC2 and Th2 cells [28], and the number of GATA3 cells was examined by IHC, which revealed that both *H*. *nana* and ESP caused an increase in GATA3 cells in the mouse intestine (Fig. 5A,C). To investigate whether IL-25 and IL-33 promote the expression of type 2 cytokines, we assessed changes in IL-13 expression level using IHC and RT-qPCR, and the expression level of IL-4 was detected by WB and RT-qPCR. The results revealed that both *H*. *nana* infection and ESP led to increased secretion of IL-13 and IL-4. The expression levels of IL-13 and IL-4 were decreased after intervention with the RCM-1 (Fig. 5B–G). The RT-qPCR result of IL-4 (Fig. 5G) is different from the WB result (Fig. 5E) and RT-qPCR detects the mRNA transcription level while WB reflects the protein level. mRNA abundance and protein abundance may not always be directly correlated because mRNA translating to protein is affected by a variety of factors, such as post-transcriptional regulation and translation efficiency of ribosome [29]. Additionally, proteins are also more stable than mRNA in vitro. The protein level is more crucial than the mRNA transcription level in such contexts. RT-qPCR analysis of the transcription levels of other type 2 cytokines, including *IL-5* and *IL-9*, results showed that RCM-1 could inhibit the increase in the transcription levels induced by* H*. *nana* infection or ESP (Fig. 5H and I). These findings indicate that *H*. *nana* infection and ESP stimulate increased the expel of type 2 cytokines. In contrast, RCM-1 causes a decrease in the expression of type 2 cytokines, which affects the expel of *H*. *nana*.

### *Hymenolepis nana* has a dual effect on the number of ISC: infection reduces and ESP promotes the proliferation, and RCM-1 intervention exacerbates the reduction of ISC

**Fig. 6:**
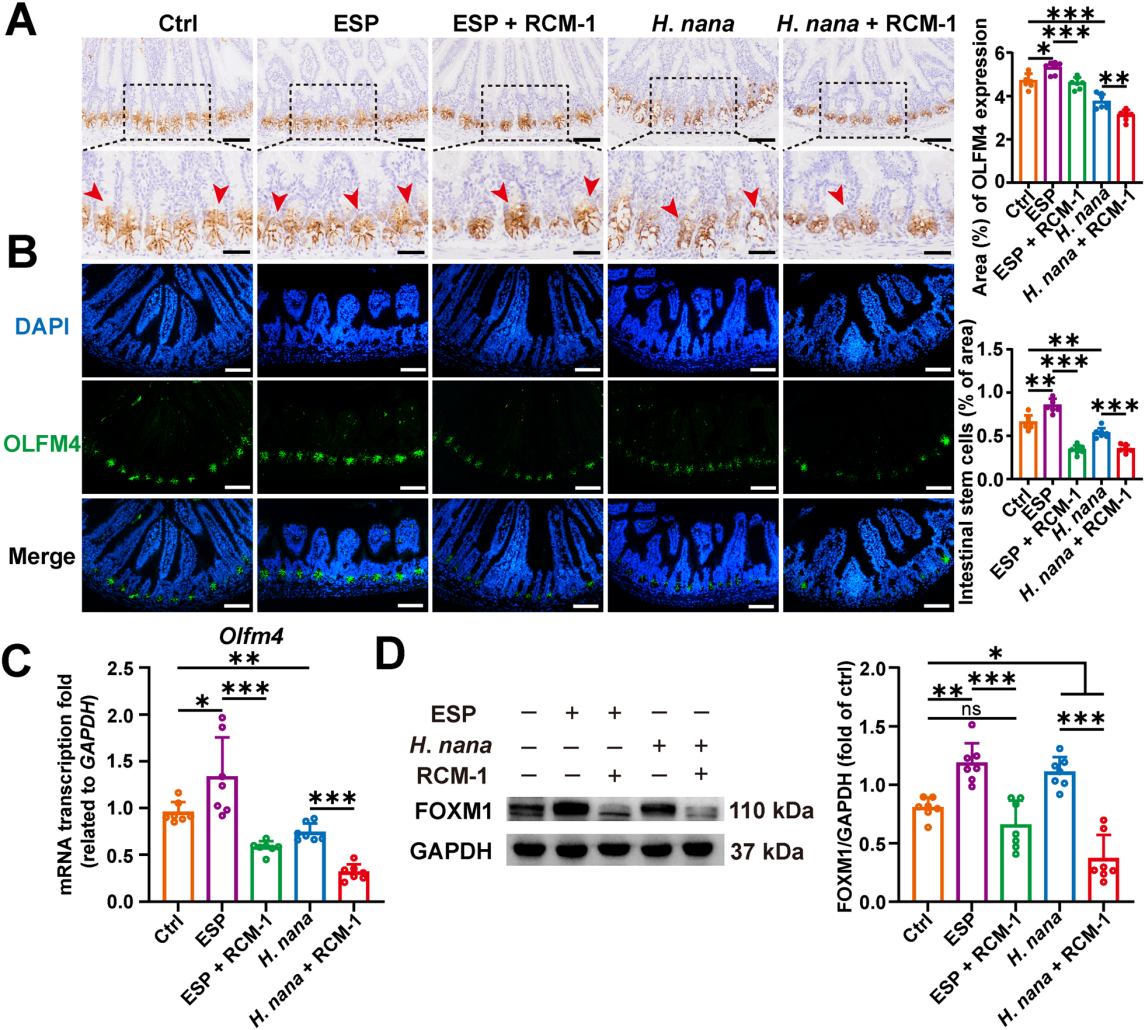
*Hymenolepis nana* infection causes a decrease in the number of ISC and ESP causes an increase in ISC; injection of RCM-1 decreases the number of ISC in mice. **A** Representative images of IHC with OLFM4 (brown highlighted by red arrowheads, scale bars 100 μm for the upper panel and 50 μm for the lower panel). **B** Representative images of IF with OLFM4 (green) and the nucleus (DAPI, blue) (scale bars 100 μm). Percentages of OLFM4-positive area and the number of ISC were semi-quantified using Image J software. **C** The transcription level of *Olfm4* and the relative quantification were determined using the 2^−ΔΔCt^ method normalized to *GAPDH*. **D** The protein level of FOXM1 (left panel) and the percentage of the relative expression of FOXM1 were semi-quantified using Image J software (right panel). Data are presented as mean + SD for (**C**) and the right panels of (**A**, **B** and **D**), *n* = 7 per group, *P < 0.05, **P < 0.01, ***P < 0.001; ns, not statistically significant

The effects of *H*. *nana* infection on the number of ISC remain unclear. To assess changes in ISC numbers, we measured the expression of the ISC marker OLFM4. The results indicated that *H*. *nana* infection leads to a reduction in ISC numbers, whereas ESP has the opposite effect, increasing ISC numbers. Additionally, changes in another ISC marker of LGR5 confirmed these findings (Additional file 4: Fig. S3). Following intervention with RCM-1, the changes in ISC numbers induced by both *H*. *nana* infection and ESP were consistent, showing a reduction (Fig. 6A–C). FOXM1 is a positive regulator of cell proliferation and has an important role in regulating the cell cycle. Our results revealed that both *H*. *nana* adult and its ESP promoted FOXM1 expression, which was inhibited by the use of RCM-1, an inhibitor of FOXM1 (Fig. 6D). This result suggests that *H*. *nana* infection may decrease ISC numbers due to mechanical damage caused by the worms, while ESP may actually promote an increase in the number of ISC by upregulating FOXM1.

### RCM-1 inhibits adult *Hymenolepis nana* and its ESP-induced increase in the number of Paneth cells, which disrupts the homeostasis of ISC

**Fig. 7:**
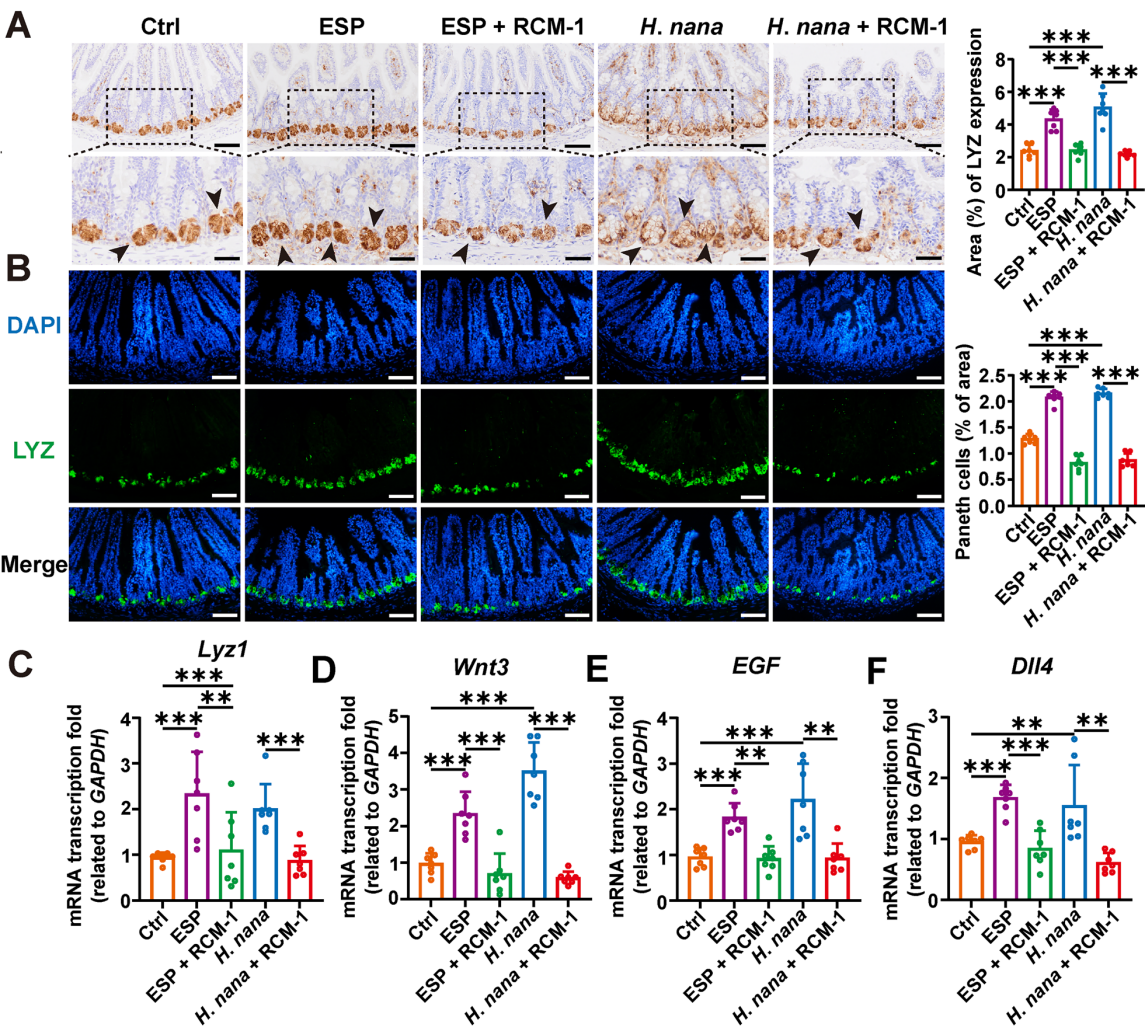
RCM-1 suppresses the increase in the number of Paneth cells induced by adult* H*. *nana* and its ESP, leading to a decrease in the secretion of antimicrobial peptides and growth factors. **A** Representative images of IHC with LYZ (brown highlighted by black arrowheads, scale bars 100 μm for the upper panel and 50 μm for the lower panel). **B** Representative images of IF with LYZ (green) and the nucleus (DAPI, blue) (scale bars 100 μm). Percentages of LYZ-positive area and the number of Paneth cells were semi-quantified using Image J software. The transcription levels of *Lyz1* (**C**), *Wnt3* (**D**), *EGF* (**E**), and *Dll4* (**F**), as well as the relative quantification, were determined using the 2^−ΔΔCt^ method normalized to *GAPDH*. Data are presented as mean + SD for (**C**–**F**) and the right panels of (**A** and **B**), *n* = 7 per group, **P < 0.01, ***P < 0.001

Paneth cells, located in the intestinal crypts, maintain ISC homeostasis by secreting related growth factors. Lysozyme (LYZ) is the first antimicrobial peptide discovered in Paneth cells and is widely used as a Paneth cell marker in the ileum [30]. Using IHC, IF, and RT-qPCR to detect LYZ, we found that both adult* H*. *nana* worms and ESP increased LYZ and the number of Paneth cells (Fig. 7A–C). Further RT-qPCR analysis of Paneth cell-secreted the transcription levels of growth factors *Wnt3*, *EGF*, and *Dll4* showed an increase (Fig. 7D–F). IL-13 can promote the secretion of antimicrobial peptides by Paneth cells, and we also observed that RCM-1 intervention resulted in a reduction in both the number of Paneth cells and the secretion of LYZ. These results indicate that *H*. *nana* promotes an increase in the number of Paneth cells, which in turn secrete growth factors to maintain ISC homeostasis. In contrast, RCM-1 decreases the number of Paneth cells, leading to decreased secretion of antimicrobial peptides and growth factors, thereby disrupting ISC homeostasis.

## Discussion

The intestine is the target organ for most parasitic infections, including both worms and protozoa. The worms typically activate type 2 immune responses in the host’s immune system and have a significant impact on the local tissue microenvironment [31]. Worms are multicellular parasites that cause a range of adverse effects, such as malnutrition and developmental delays in children, contributing to the global public health burden [32]. Intestinal worms have evolved mechanisms to evade or suppress host defense responses to ensure their survival and reproduction, laying eggs in the intestinal tissues and being expelled through the host’s feces [33]. *Hymenolepis nana* (*H*. *nana*), also known as the dwarf tapeworm, primarily parasitizes the intestines of humans and rodents. *H. nana* has a global distribution, with an estimated infection rate of 1.2% in humans and up to 13% in rodents [21]. Conventional diagnostic methods for hymenolepiasis caused by *H*. *nana* involve fecal examination of eggs or adults. However, PCR-based molecular techniques can improve parasite detection rates and accurately determine species differentiation and genetic characteristics. Mitochondrial cytochrome c oxidase subunit I (*COX-I*) is an important mitochondrial gene with high variability and specificity among different parasites, making it one of the most commonly used molecular genetic markers [34].

Intestinal worms parasitize the host’s intestines, causing a series of damages, including nutrient depletion, mechanical damage from the worm’s body, and toxic effects from ESP [33]. In the current study, we identified *H*. *nana* through morphological and molecular biology techniques and assessed the quantity and molecular size of the proteins contained in ESP by immunoblotting. We then explored the immune response relationship between *H*. *nana* and the host using *H*. *nana* egg infections and intraperitoneal ESP injections, and whether RCM-1 affected the immune response to *H*. *nana* in mice. Our findings showed that adult *H*. *nana* worms and ESP could lead to an increase in the number of goblet cells, tuft cells, and Paneth cells, as well as an increase in type 2 cytokine secretion, ultimately aimed at clearing *H*. *nana*. When RCM-1 was used, the host failed to produce effective immune responses to *H*. *nana*, thereby exacerbating *H*. *nana* infection in the host.

Infection with *H*. *nana* in mice can lead to degeneration or destruction of the normal villous structure of the small intestine epithelium, resulting in villous shortening or atrophy [35]. Infection of rats with *H*. *nana* results in enteritis, villous necrosis, and inflammatory infiltration of the mucosa and submucosa [36]. MUC2 is primarily secreted by goblet cells, forming a mucosal barrier that protects the mucosal surface and plays a crucial role in regulating intestinal immune responses, maintaining mucosal health, and protecting the immune system [37]. Our study found that both adult* H*. *nana* worms and ESP caused the shortening of intestinal villi and an increase in mucin MUC2 secretion in mice. Intervention with the RCM-1 could restore villous shortening induced by ESP but exacerbated villous shortening caused by adult *H*. *nana* worms. This might be due to RCM-1 increasing the severity of* H*. *nana* infection, thereby intensifying damage to the host. The above results indicated that both adult *H*. *nana* and ESP caused the shortening of intestinal villi, and the greater the worm infection, the more severe the damage.

Tuft cells occupy a small proportion of the intestinal epithelium but play a crucial role in recognizing and responding to parasitic infections [38]. It has been observed that the number of tuft cells in the small intestine increases after intestinal parasite infection. Normally, tuft cells constitute only about 1% of the intestinal epithelial cells in mice, but their numbers can increase several-fold during parasitic infections [39, 40]. Tuft cells have become key sentinels in parasitic infections by releasing alarmin IL-25, which activates ILC2 and enhances the type 2 immune responses, signaling the presence of worms [12]. The type 2 immune responses are protective for the host, not only by directly killing parasites in tissues or reducing their numbers by expulsion from the intestine, but also by protecting the host from tissue damage caused by large extracellular parasites migrating within the body [41]. The regulation of signaling pathways between tuft cells and ILC2 has garnered significant attention in parasitic intestinal infections. Tuft cells can recognize infections from *Nippostrongylus brasiliensis*, *Trichinella spiralis*, and *Helicotylenchus*, releasing IL-25 to increase ILC2 numbers and subsequently IL-13 secretion, which acts on ISC to differentiate into more tuft and goblet cells [12, 39, 42]. IL-33 is a tissue-derived nuclear cytokine that promotes type 2 immune responses during allergy and helminthic infection [43]. Our study found that both adult* H*. *nana* worms and ESP induced increases in the numbers of goblet and tuft cells, along with elevated secretion of cytokines IL-25 and IL-33. Further investigation into the expression changes of type 2 cytokines revealed an increase in type 2 cytokines. The above series of changes could be inhibited by RCM-1, indicating that adult *H*. *nana* worms and ESP could activate the type 2 immune response, and RCM-1-induced reduction in type 2 cytokines secretion affected host excretion of *H*. *nana*.

FOXM1 plays an important role in the cell cycle and promotes the renewal of ISC [15]. ISC are located in the intestinal crypts and possess high proliferative and differentiative capacities. The proliferation and differentiation of ISC ensure the integrity and functional stability of the intestinal epithelium and contribute to the prevention of intestinal diseases [44]. A recent study has shown that ISC depletion occurs locally in mice infected with *Heligmosomoides polygyrus* [45]. ISC can differentiate into mature Paneth cells; unlike other mature intestinal epithelial cells, they migrate downward after differentiation and reside at the base of the crypts, where they are arranged in a cross pattern with ISC [46]. Following the parasitic invasion of the host intestine, ISC accelerate their proliferation and differentiation, stimulating the proliferation of Paneth cells in response to the infection [47]. Paneth cells can secrete antimicrobial peptides to maintain intestinal homeostasis [48] and regulate ISC function through the secretion of growth factors such as Wnt3, EGF, and Dll4 [49]. Research on the impact of intestinal worm infections on host small intestinal Paneth cells is relatively limited. The study has shown that *T. spiralis* and *N. brasiliensis* infections cause a significant increase in Paneth cell numbers [50]. In addition, IL-13 promotes ISC proliferation and differentiation on the one hand and enhances the secretion of antimicrobial peptides by Paneth cells on the other [51]. The effects of *H*. *nana* infection on ISC and Paneth cells remain unclear. Our research findings indicated that the adults of *H*. *nana* reduced the number of ISC, and RCM-1 exacerbated this effect, likely due to mechanical damage caused by *H*. *nana* adults in the intestinal lumen. ESP increased the number of ISC, but blocking the IL-13/STAT6 signaling pathway and FOXM1 resulted in a decrease in the number of ISC, suggesting that ESP-induced upregulation of IL-13 and FOXM1 may promote ISC proliferation. Both *H*. *nana* adults and ESP increased lysozyme and Paneth cell numbers, consistent with IL-13 changes. This suggested that Paneth cells secrete signaling factors to maintain ISC homeostasis and that IL-13 also promoted Paneth cell proliferation, which increased lysozyme secretion and maintained intestinal homeostasis.

Collectively, we found that both *H*. *nana* adult and its ESP promoted ISC differentiation into goblet cells, tuft cells, and Paneth cells, as well as inducing an increase in type 2 cytokines. This finding suggested that the infection with *H*. *nana* triggered the activation of the host’s tuft/IL-13 and FOXM1 signaling pathways, eliciting the immune responses that facilitated the expulsion of *H*. *nana* from the host.

## Conclusions

In this study, we found that *H*. *nana* adult and its ESP could activate the tuft/IL-13 and FOXM1 signaling pathways in the mouse intestine, leading to the differentiation of ISC into more goblet and tuft cells, as well as an increase in Paneth cells to maintain the stability of ISC and activate the type 2 immune response in mice. These changes helped the host to expel the parasites. In contrast, RCM-1 suppressed the host’s immune responses to *H*. *nana*, resulting in the host’s inability to effectively kill or expel *H*. *nana*. This study provided preliminary insights into the role of intestinal epithelial cells in defense against *H*. *nana* and explored the relationship between *H*. *nana* and the host’s immune responses, providing a foundation for the prevention and treatment of *H*. *nana* and its helminth therapy.

## Supplementary Information

Additional file 1: Supplementary reagents and methods.

Additional file 2: Figure S1. Effects of different doses of ESP on mouse intestinal goblet cells and tuft cells. ESP1 indicates an intraperitoneal dose of 25 μg/day per mouse and ESP2 indicates a dose of 50 μg/day per mouse. (A) Representative images of AB-PAS-stained with the goblet cells (sharp or deep blue pointed by red arrowheads, scale bars 100 μm for the upper panel, and 50 μm for the lower panel). (B) Representative images of IF with MUC2 (green) and the nucleus (DAPI, blue) (scale bars 100 μm). (C) Representative images of IHC with DCLK1 (brown pointed by black arrowheads, scale bars 100 μm for the upper panel, and 50 μm for the lower panel). (D) Representative images of IF with DCLK1 (green) and the nucleus (DAPI, blue) (scale bars 100 μm). Percentages of the statistics of AB-PAS-stained positive area (E), and the number of goblet cells (F), tuft cells (H), and DCLK1-positive area (G) were semi-quantified using Image J software. Data are presented as mean + SD for (E)–(H), *n* = 6 per group, ** *P* < 0.01, *** *P* < 0.001.

Additional file 3: Figure S2. The identification of *H. nana* and ESP. (A) Representative picture of the hamsters from an urban pet market. (B) Representative image of the egg of *H*. *nana* (scale bar 20 μm). (C) Representative image of the scolex of *H*. *nana*, the sucker and the restellum are indicated by the black and red arrowheads respectively (scale bar 100 μm). (D) Representative image of mature proglottids of *H*. *nana* (scale bar 100 μm). (E) Representative image of gravid proglottids of *H. nana* (scale bar 100 μm). (F) PCR amplification electrophoresis of the COX-I of *H*.  *nana*, the proposed amplicon was 202 bp. M: DL 2000 marker, Lane 1: The genomic DNA of *H*.* nana *as the PCR template, Lane 2: sterilized H2O as the PCR template. (G) The egg of *H*.  *nana* (detected from the feces of mice) (scale bar 20 μm). (H) Adult worms were dissected from the intestines of *H*. *nana*-infected mice. (I) Immunoblotting result of ESP. M: protein marker; Lane 1-4: ESP.

Additional file 4: Figure S3. *H*. *nana* infection causes a decrease in the number of ISC and ESP causes an increase in ISC. (A) Representative images of IF with LGR5 (green) and the nucleus (DAPI, blue) (scale bars 100 μm). (B) The protein level of LGR5. Percentages of the number of ISC (C) and the relative expression of LGR5 (D) were semi-quantified using Image J software. (E) The transcription level of *Lgr5* and the relative quantification were determined using the 2^-ΔΔCt^ method normalized to *GAPDH*. Data are presented as mean + SD for (C)–(E), *n* =7 per group for (C), *n* = 6 per group for (D)–(E), ** *P* < 0.01, *** *P* < 0.001.

Additional file 5: Table S1. Primer sequences used for RT-qPCR experments of the current study.

## Data Availability

Data are provided within the manuscript or supplementary information files.

## Abbreviations

*H*. *nana*: *Hymenolepis nana*
ESP: Excretory–secretory products
ILC: Innate lymphoid cells
ISC: Intestinal stem cells
EGF: Epidermal growth factor
H&E: Hematoxylin and eosin
AB-PAS: Alien-blue and periodic acid-Schiff
RT-qPCR: Real-time quantitative polymerase chain reaction
IHC: Immunohistochemistry
IF: Immunofluorescence
WB: Western blotting
LYZ: Lysozyme
*COX-I*: Cytochrome c oxidase subunit I
