# Supplementary material for: Adult Hymenolepis nana and its excretory–secretory products elicit mouse immune responses via tuft/IL-13 and FOXM1 signaling pathways
Source: Parasit Vectors. 2025 Mar 11;18:100. doi: 10.1186/s13071-025-06719-w (PMC11899370; doi:10.1186/s13071-025-06719-w)
Supplement: Supplementary file 1 — Additional file 1: Supplementary reagents and methods. [file 13071_2025_6719_MOESM1_ESM.docx]

**Supplementary materials for**

Adult *Hymenolepis nana* and its excretory-secretory products elicit mouse immune responses via Tuft/IL-13 and FOXM1 signalling pathways

Rong Mou, Xuan-Yin Cui, Yu-Si Luo, Yi Cheng, Qing-Yuan Luo, Zhen-Fen Zhang, Wen-Lan Wu, Jin-Fu Li, Ke Zhang

Email: kezhang@gmc.edu.cn (KZ)

**Reagents and methods**

**1.** **Reagents**

Hematoxylin-Eosin Stain Kit (G1120) was offered by Solarbio (Beijing, China). AB-PAS Stain Kit (R22021) was obtained from Saint-Bio (Shanghai, China). DAB staining kit (ZLI-9018, zsbio), EDTA antigen retrieval solution (C1034), Penicillin / Streptomycin / Amphotericin B (sterile solution) (P7630), and carmine (1390-65-4, Macklin) were offered by Guiyang Jingong Technology Co. (Guiyang, China). Real-time quantitative PCR (RT-qPCR) Reverse Transcription Kit (11141ES60), SYBR RT-qPCR Kit (11201ES08), Super enhanced chemiluminescence (ECL) Detection Reagent (36208ES60), TRIeasy Total RNA Extraction Reagent (10606ES60), RIPA lysis buffer (20101ES60), BCA Protein Quantification Kit (20201ES76), MolPure Cell / Tissue DNA Kit (18700ES50), and RPMI1640 medium (41402ES76) were provided by Yeasen (Shanghai, China). Goat serum (abs933) was purchased from Absin (Shanghai, China). RCM-1 (S6898) was offered by Selleck (Shanghai, China). Trichloromethane, isopropanol, and ethanol absolute are analytically pure reagents.

For western blotting (WB), primary antibodies for LGR5 (R380973, rabbit) was obtained from ZenBio (Chengdu, China), for IL-4 (66142-1-lg, mouse) and FOXM1 (13147-1-AP, rabbit) were offered by Proteintech (Wuhan, China), and for GAPDH (AF7021, rabbit) was purchased from Affinity (Jiangsu, China). Secondary antibodies (HY-P8001, anti-rabbit) and (HY-P8004, anti-mouse) for WB and immunohistochemistry (IHC) were purchased from MCE (Shanghai, China). For immunofluorescence (IF) or IHC, primary antibodies for IL-13 (A00077-2, rabbit) was offered by Boster (Wuhan, China), for GATA3 (ER1901-20, rabbit) was provided by HuaBio (Hangzhou, China), and for MUC2 (ab272692, rabbit), lysozyme (LYZ) (ab108508, rabbit), and DCLK1 (ab31704, rabbit) were obtained from Abcam (Cambridge, UK), for OLFM4 (39141, rabbit) was purchased from Cell Signaling Technology (Danvers, MA, USA), for LGR5 (DF2816, rabbit) was purchased from Affinity (Jiangsu, China). Fluorescent secondary antibody anti-rabbit 488 (A21206) was offered by Thermo Fisher Scientific (Waltham, MA, USA).

**2. Identification of *H*. *nana* and ESP, and the acquisition of serum from *H*. *nana-*infected hamster**

A few parasites were randomly selected and cut with a scissor to obtain eggs. In addition, six parasites were randomly selected from those obtained and stained with carbolic acid red (configured with carmine), eggs and stained adults were observed using the fluorescence microscope (Eclipse 80i, Nikon Ltd., Japan). A few parasites were re-selected and worm DNA was extracted using a MolPure Cell / Tissue DNA Kit (Yeasen, China), and PCR amplification for the *COX-I* gene of *H*. *nana* followed by agarose gel electrophoresis. The *COX-I* primer sequence used in this process is listed in Additional file 5: Table S1. Microscopic examination revealed that the eggs were nearly spherical with a relatively thin eggshell, containing a thicker embryonic membrane within, and inside the membrane, an oncosphere with visible small hooks was observed (Additional file 3: Figure S2B). After staining the adult worms with carboxyl borate red, the entire worm body appeared red, with the scolex showing a rostellum with distinct hooks, as well as visible circular suckers (Additional file 3: Figure S2C). The mature proglottid exhibited reproductive systems such as testes and ovaries (Additional file 3: Figure S2D), and the gravid proglottids were filled with eggs (Additional file 3: Figure S2E). PCR amplification and agarose gel electrophoresis of the *H*. *nana* *COX-I* gene showed a clear band at 202 bp (Additional file 3: Figure S2F). Combined with the above results, we confirmed that the parasites obtained from the hamster were *H*. *nana*.

For serum from *H*. *nana*-infected hamsters, peripheral blood samples were obtained, stood at room temperature for 2 h, centrifuged at 10,000 x g for 10 min, then aspirated the serum, and stored at -80°C.

In parallel, the protein composition of the ESP was scrutinized through immunoblotting. In brief, equal quantities of ESP samples were loaded into each well, and electrophoresis was performed. The resolved proteins were then transferred onto a PVDF membrane, which was blocked with 5% skim milk to prevent nonspecific antibody interactions. The membrane was probed overnight with a primary antibody (serum from the *H*. *nana*-infected hamster, diluted 1:50), followed by the addition of an anti-mouse secondary antibody (diluted 1:10,000) and a 1 h incubation. Finally, the protein bands were visualized using Super ECL Detection Reagent, allowing for the assessment of the number and molecular size of proteins present in the ESP. The ESP harvested after culture was analyzed by immunoblotting, revealing a variety of proteins, with the main bands located at 20-25kDa, 35-45kDa, 45-60kDa, and 100-140kDa, respectively (Additional file 3: Figure S2I).

**3. Extraction of tissues total RNA**

Extract total RNA from mouse ileum tissues with 1 ml TRIeasy Total RNA Extraction Reagent (Yeasen, China), then add 200 μl trichloromethane, and centrifuge at 4°C 13,000 x g for 20 min, take the supernatant, add an equal amount of isopropanol, mix well and centrifuge at 4°C 13,000 x g for 10 min, discard the supernatant, and the precipitate at the bottom will be total RNA, add 75% ethanol to wash the RNA precipitate, centrifuge at 4°C 5,400 x g for 10 min, and finally add 30-50 μl DEPC water.

**4. Ileum hematoxylin-eosin (H&E) staining and Alien-blue and Periodic acid-Schiff (AB-PAS) staining**

The ileum tissues of the mice were collected and fixed in 4% paraformaldehyde, embedded in paraffin, and cut into 4 µm thick sections. Treated the sections with xylene I for 15 min and xylene II for 15 min; followed by 100% ethanol I, 100% ethanol II, 95% ethanol I, 95% ethanol II, and 75% ethanol for 2 min each. According to the reagent instructions, H&E staining to observe changes in the small intestinal epithelial villi and AB-PAS staining for ileum goblet cells were performed. After sealing the slices with neutral gum, images were observed and captured using a slide scanner (Olympus SLIDEVIEW VS200, Japan).

**5. Semi-quantitative evaluation of stained areas using Image J**

For cell counting of AB-PAS staining slide, the stained images were acquired and input into Image J (version 1.53i, US National Institutes of Health, USA), converting them to an 8-bit format, adjusting the threshold to separate the stained cells from the background, utilizing tool “Analyze Particles” to automatically count the cells based on size, shape, and intensity parameters, and finally reviewing the results manually to ensure accuracy, with additional steps such as using “Fill Holes” and “Watershed” to refine cell detection in cases of overlapping or irregularly shaped cells. Finally, the collected data was compiled and analyzed statistically.

To perform the semi-quantitative assessment for IHC, IHC-stained tissue section images were first acquired and prepared in Image J (version 1.53i, US National Institutes of Health, USA), including converting to grayscale and separating the staining color from the background using color deconvolution. Next, thresholding was applied to create a binary image, and the stained area was measured using the “Analyze Particles” feature. Staining intensity could be quantified by calculating the mean gray value or integrated density of the stained areas. Finally, the collected data was compiled and analyzed statistically.

To perform the semi-quantitative assessment of IF result, images of stained tissue sections were first acquired and imported into Image J (version 1.53i, US National Institutes of Health, USA). The images were then processed to enhance contrast and clarity, and regions of interest (ROIs) were selected. Next, the staining intensity within the ROIs was measured using Image J's built-in analysis tools, such as the “Measure” function, which provided metrics such as mean gray value and integrated density. Finally, the collected data was compiled and analyzed statistically to quantify the IF staining intensity and assess differences between samples.

**Figure S1.** **Effects of different doses of ESP on mouse intestinal goblet cells and tuft cells.** ESP1 indicates an intraperitoneal dose of 25 μg/day per mouse and ESP2 indicates a dose of 50 μg/day per mouse. (**A**) Representative images of AB-PAS-stained with the goblet cells (sharp or deep blue pointed by red arrowheads, scale bars 100 μm for the upper panel, and 50 μm for the lower panel). (**B**) Representative images of IF with MUC2 (green) and the nucleus (DAPI, blue) (scale bars 100 μm). (**C**) Representative images of IHC with DCLK1 (brown pointed by black arrowheads, scale bars 100 μm for the upper panel, and 50 μm for the lower panel). (**D**) Representative images of IF with DCLK1 (green) and the nucleus (DAPI, blue) (scale bars 100 μm). Percentages of the statistics of AB-PAS-stained positive area (**E**), and the number of goblet cells (**F**), tuft cells (**H**), and DCLK1-positive area (**G**) were semi-quantified using Image J software. Data are presented as mean + SD for (E)-(H), *n* = 6 per group, ** *p* < 0.01, *** *p* < 0.001.

**Figure S2. The identification of *H*. *nana* and ESP.** (**A**) Representative picture of the hamsters from an urban pet market. (**B**) Representative image of the egg of *H*. *nana* (scale bar 20 μm). (**C**) Representative image of the scolex of *H*. *nana*, the sucker and the restellum are indicated by the black and red arrowheads respectively (scale bar 100 μm). (**D**) Representative image of mature proglottids of *H*. *nana* (scale bar 100 μm). (**E**) Representative image of gravid proglottids of *H*. *nana* (scale bar 100 μm). (**F**) PCR amplification electrophoresis of the *COX-I* of *H*. *nana*, the proposed amplicon was 202 bp. M: DL 2000 marker, Lane 1: The genomic DNA of *H*. *nana* as the PCR template, Lane 2: sterilized H_2_O as the PCR template. (**G**) The egg of *H*. *nana* (detected from the feces of mice) (scale bar 20 μm). (**H**) Adult worms were dissected from the intestines of *H*. *nana*-infected mice. (**I**) Immunoblotting result of ESP. M: protein marker; Lane 1-4: ESP.


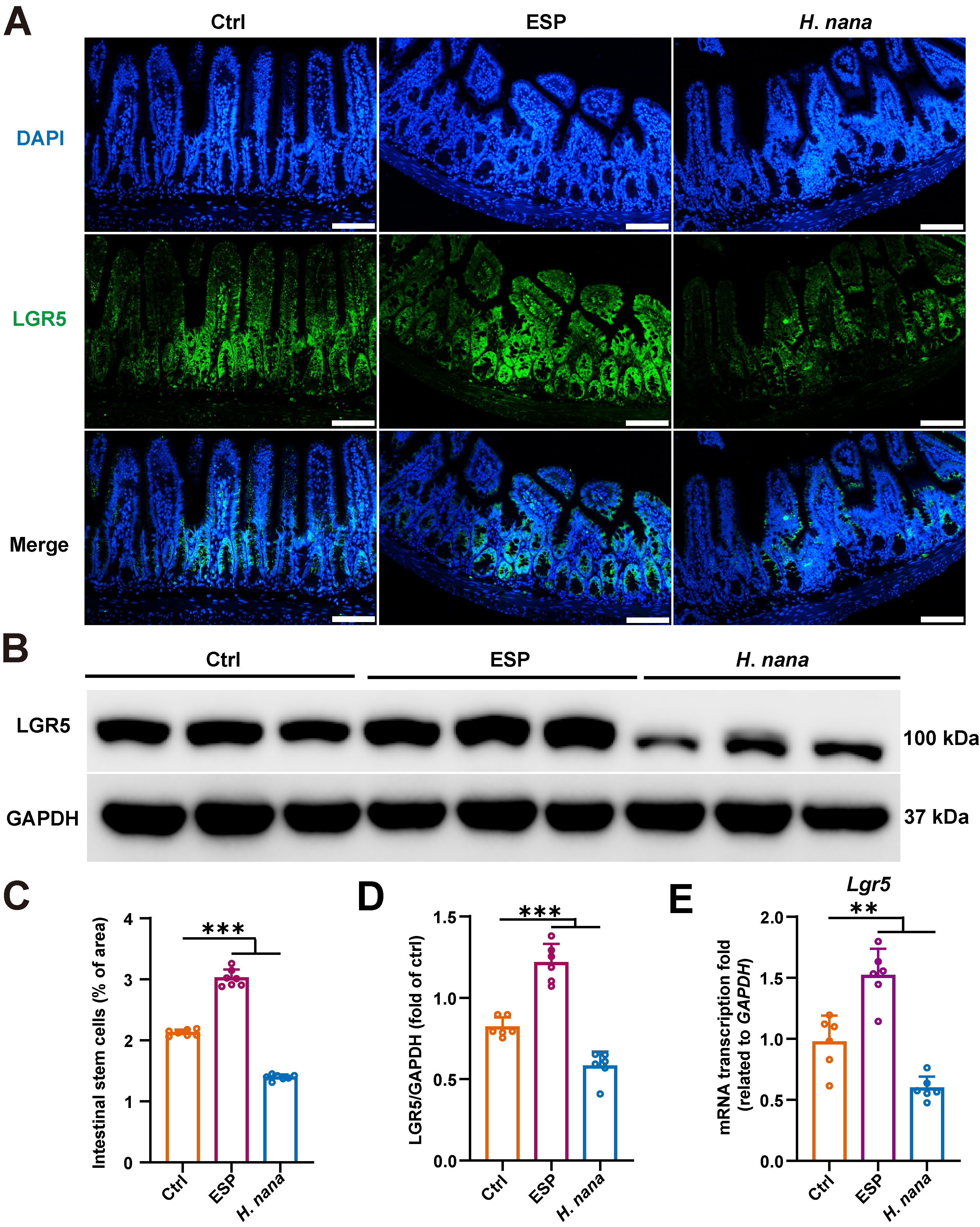


**Figure S3. *H*. *nana* infection causes a decrease in the number of ISC and ESP causes an increase in ISC.** (**A**) Representative images of IF with LGR5 (green) and the nucleus (DAPI, blue) (scale bars 100 μm). (**B**) The protein level of LGR5. Percentages of the number of ISC (**C**) and the relative expression of LGR5 (**D**) were semi-quantified using Image J software. (**E**) The transcription level of *Lgr5* and the relative quantification were determined using the 2^-ΔΔCt^ method normalized to *GAPDH*. Data are presented as mean + SD for (C)-(E), *n* =7 per group for (C), *n* =6 per group for (D)-(E), ** *p* < 0.01, *** *p* < 0.001.

**Table S1. Primer sequences used for RT-qPCR experiments of the current study**

| Target Genes | Primer Sequences (5’ - 3’) |
| --- | --- |
| *COX-I* | F: ACCGCGTCGTGTGTGTATTT  R: ACATGCAA CTGGGCTCATACG |
| *Lgr5* | F: CCTGGGAAAGCATACCCGTT  R: GGTTGACTCACAGGACCGTT |
| *Olfm4* | F: ACACAGCTCACATCCTTTCTC  R: GATGCTGTCCTTCTCCATGAC |
| *Lyz1* | F: CCCAAGATCTAAGAATGCCTGT  R: CCCATGCTCGAATGCCTT |
| *Wnt3* | F: GCTGCCAAGAGTGTATTCGC  R: CCGCACAATCTACCCCTTCC |
| *EGF* | F: GTGGCTCCGTCCGTCTTATC  R: GGCTATCCAAATCGCCTTGC |
| *Dll4* | F: AAGGTGCCACTTCGGTTACA  R: GGCAATCACACACTCGTTCC |
| *Dclk1* | F: CAGCCTGGACGAGCTGGTGG  R: TGACCAGTTGGGGTTCACAT |
| *Muc2* | F: ACCACAATCTCTACTCCCATCT  R: TCCAGTCAGACCAAAAGCAG |
| *IL-25* | F: CAGCCTGGACGAGCTGGTGG  R: TGACCAGTTGGGGTTCACAT |
| *IL-33* | F: GTATTCCAACTCCAAGATTTCCC  R: CATGCAGTAGACATGGCAGA |
| *IL-4* | F: GCTCGTCTGTAGGGCTTCC  R: GTGCAGCTTATCGATGAATCCAG |
| *IL-5* | F: GACAAGCAACGAGACGGTGA  R: TTGGAATAGCGTCTCCACGG |
| *IL-9* | F: TGCTCTTCAGTTCTGTGCTGG  R: GACGGAGAGACACAAGCAGC |
| *IL-13* | F: AGCTCCCTGGTTCTCTCACT  R: CTCATTAGAAGGGGCCGTGG |
| *GAPDH* | F: AGGAGCGAGACCCCACTAACA  R: AGGGGGGCTAAGCAGTTGGT |
