# Supplementary figures and images for: Adult Hymenolepis nana and its excretory–secretory products elicit mouse immune responses via tuft/IL-13 and FOXM1 signaling pathways
Source: Parasit Vectors. 2025 Mar 11;18:100. doi: 10.1186/s13071-025-06719-w (PMC11899370; doi:10.1186/s13071-025-06719-w)

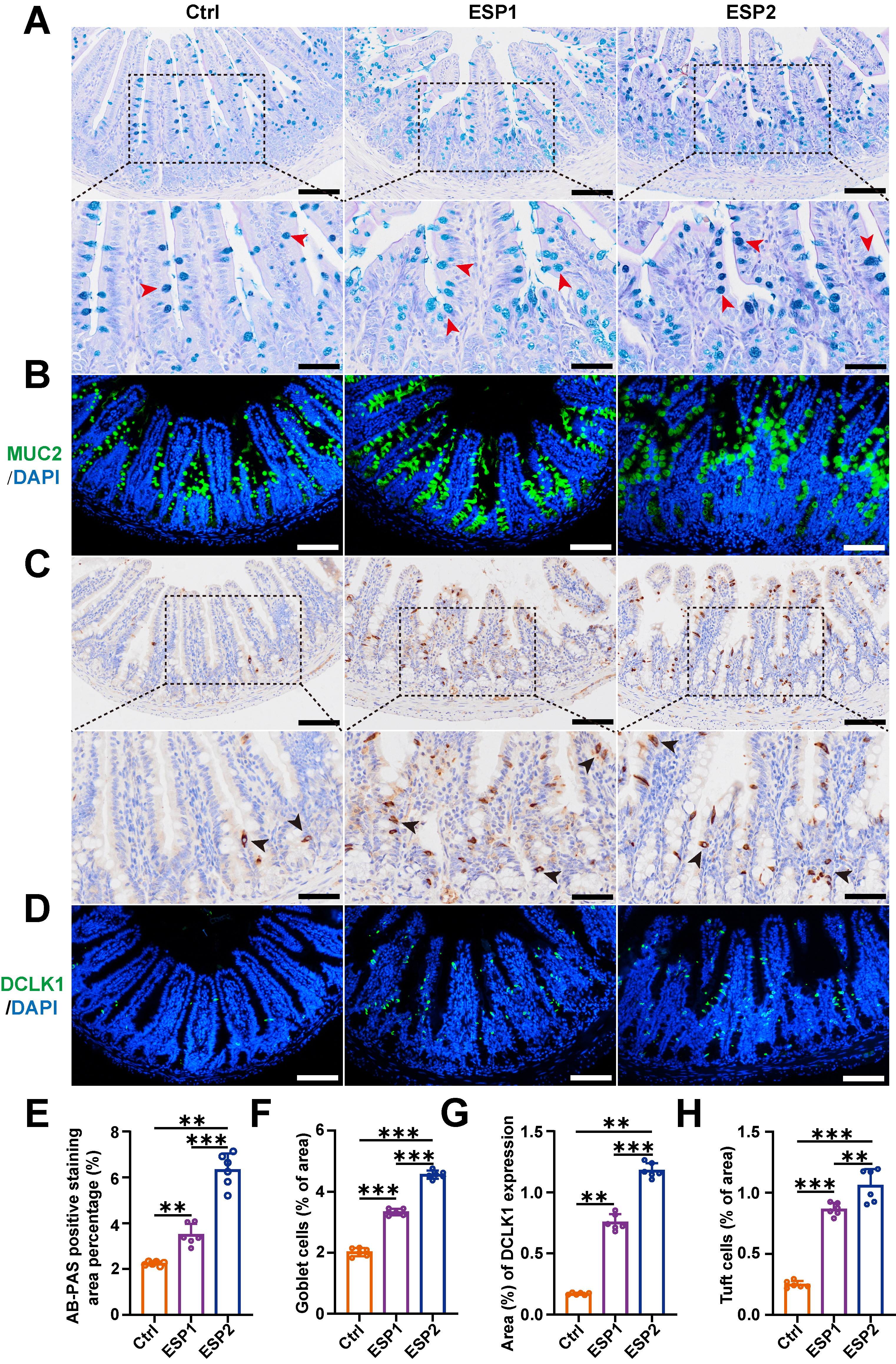

Supplement: Supplementary file 2 — Additional file 2: Figure S1. Effects of different doses of ESP on mouse intestinal goblet cells and tuft cells. ESP1 indicates an intraperitoneal dose of 25 μg/day per mouse and ESP2 indicates a dose of 50 μg/day per mouse. (A) Representative images of AB-PAS-stained with the goblet cells (sharp or deep blue pointed by red arrowheads, scale bars 100 μm for the upper panel, and 50 μm for the lower panel). (B) Representative images of IF with MUC2 (green) and the nucleus (DAPI, blue) (scale bars 100 μm). (C) Representative images of IHC with DCLK1 (brown pointed by black arrowheads, scale bars 100 μm for the upper panel, and 50 μm for the lower panel). (D) Representative images of IF with DCLK1 (green) and the nucleus (DAPI, blue) (scale bars 100 μm). Percentages of the statistics of AB-PAS-stained positive area (E), and the number of goblet cells (F), tuft cells (H), and DCLK1-positive area (G) were semi-quantified using Image J software. Data are presented as mean + SD for (E)–(H), n = 6 per group, ** P < 0.01, *** P < 0.001. [file 13071_2025_6719_MOESM2_ESM.tif]

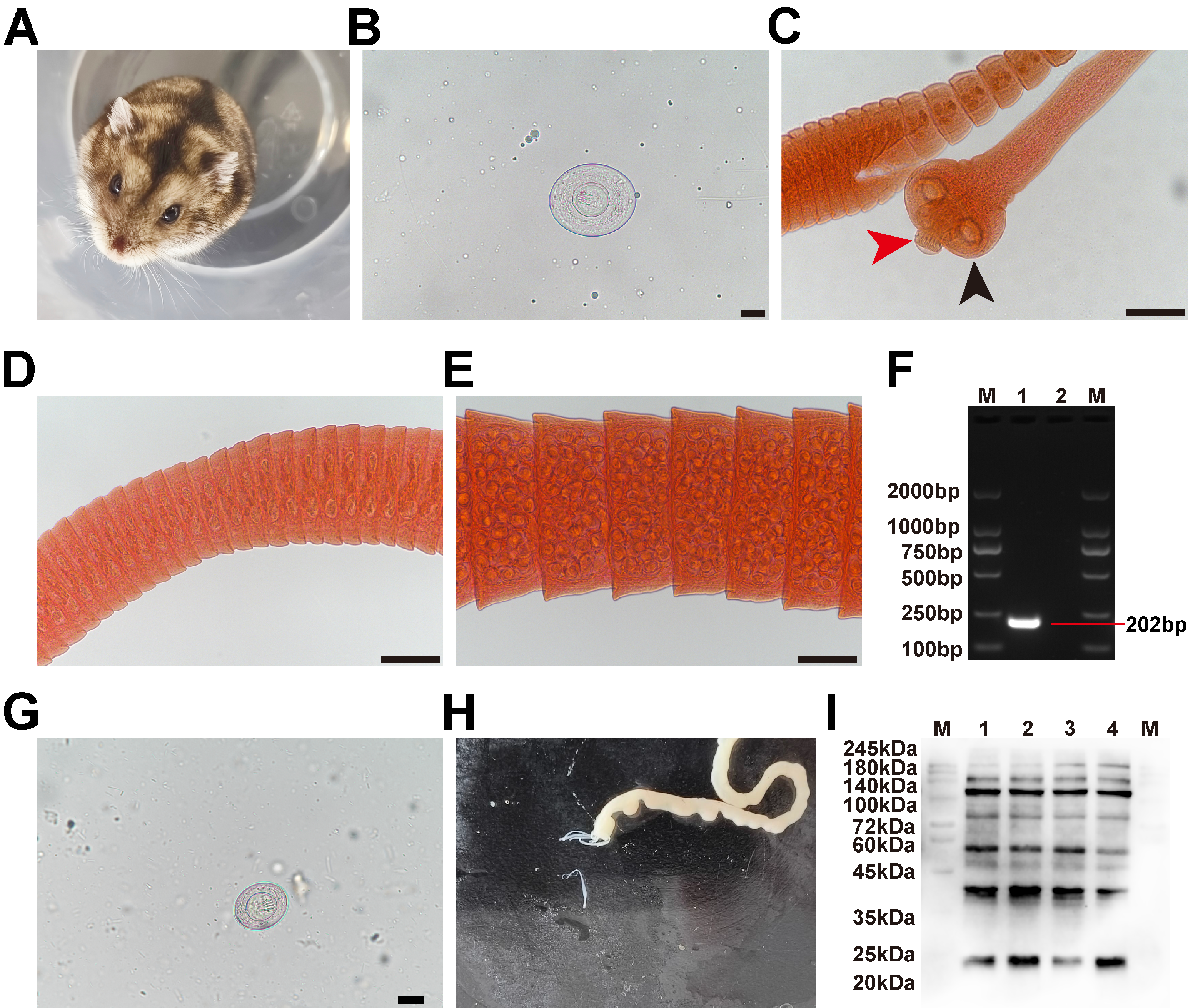

Supplement: Supplementary file 3 — Additional file 3: Figure S2. The identification of H. nana and ESP. (A) Representative picture of the hamsters from an urban pet market. (B) Representative image of the egg of H. nana (scale bar 20 μm). (C) Representative image of the scolex of H. nana, the sucker and the restellum are indicated by the black and red arrowheads respectively (scale bar 100 μm). (D) Representative image of mature proglottids of H. nana (scale bar 100 μm). (E) Representative image of gravid proglottids of H. nana (scale bar 100 μm). (F) PCR amplification electrophoresis of the COX-I of H. nana, the proposed amplicon was 202 bp. M: DL 2000 marker, Lane 1: The genomic DNA of H. nana as the PCR template, Lane 2: sterilized H2O as the PCR template. (G) The egg of H. nana (detected from the feces of mice) (scale bar 20 μm). (H) Adult worms were dissected from the intestines of H. nana-infected mice. (I) Immunoblotting result of ESP. M: protein marker; Lane 1-4: ESP. [file 13071_2025_6719_MOESM3_ESM.tif]

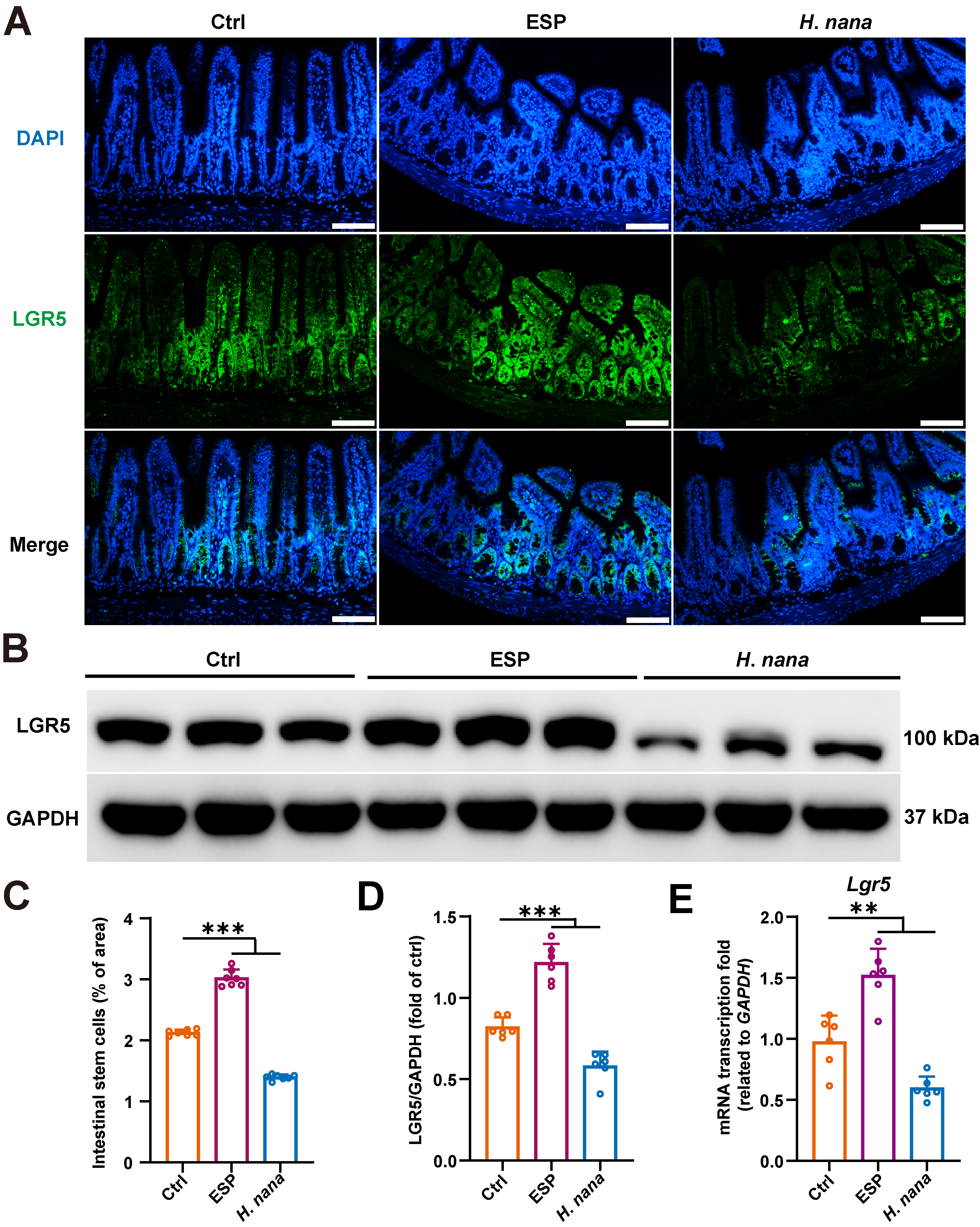

Supplement: Supplementary file 4 — Additional file 4: Figure S3. H. nana infection causes a decrease in the number of ISC and ESP causes an increase in ISC. (A) Representative images of IF with LGR5 (green) and the nucleus (DAPI, blue) (scale bars 100 μm). (B) The protein level of LGR5. Percentages of the number of ISC (C) and the relative expression of LGR5 (D) were semi-quantified using Image J software. (E) The transcription level of Lgr5 and the relative quantification were determined using the 2-ΔΔCt method normalized to GAPDH. Data are presented as mean + SD for (C)–(E), n =7 per group for (C), n = 6 per group for (D)–(E), ** P < 0.01, *** P < 0.001. [file 13071_2025_6719_MOESM4_ESM.tif]
