# Supplementary material for: Adult Hymenolepis nana and its excretory–secretory products elicit mouse immune responses via tuft/IL-13 and FOXM1 signaling pathways
Source: Parasit Vectors. 2025 Mar 11;18:100. doi: 10.1186/s13071-025-06719-w (PMC11899370; doi:10.1186/s13071-025-06719-w)
Supplement: Supplementary file 5 — Additional file 5: Table S1. Primer sequences used for RT-qPCR experments of the current study. [file 13071_2025_6719_MOESM5_ESM.docx]

**Table S1. Primer sequences used for RT-qPCR experiments of the current study**

| Target Genes | Primer Sequences (5’ - 3’) |
| --- | --- |
| *COX-I* | F: ACCGCGTCGTGTGTGTATTT  R: ACATGCAA CTGGGCTCATACG |
| *Lgr5* | F: CCTGGGAAAGCATACCCGTT  R: GGTTGACTCACAGGACCGTT |
| *Olfm4* | F: ACACAGCTCACATCCTTTCTC  R: GATGCTGTCCTTCTCCATGAC |
| *Lyz1* | F: CCCAAGATCTAAGAATGCCTGT  R: CCCATGCTCGAATGCCTT |
| *Wnt3* | F: GCTGCCAAGAGTGTATTCGC  R: CCGCACAATCTACCCCTTCC |
| *EGF* | F: GTGGCTCCGTCCGTCTTATC  R: GGCTATCCAAATCGCCTTGC |
| *Dll4* | F: AAGGTGCCACTTCGGTTACA  R: GGCAATCACACACTCGTTCC |
| *Dclk1* | F: CAGCCTGGACGAGCTGGTGG  R: TGACCAGTTGGGGTTCACAT |
| *Muc2* | F: ACCACAATCTCTACTCCCATCT  R: TCCAGTCAGACCAAAAGCAG |
| *IL-25* | F: CAGCCTGGACGAGCTGGTGG  R: TGACCAGTTGGGGTTCACAT |
| *IL-33* | F: GTATTCCAACTCCAAGATTTCCC  R: CATGCAGTAGACATGGCAGA |
| *IL-4* | F: GCTCGTCTGTAGGGCTTCC  R: GTGCAGCTTATCGATGAATCCAG |
| *IL-5* | F: GACAAGCAACGAGACGGTGA  R: TTGGAATAGCGTCTCCACGG |
| *IL-9* | F: TGCTCTTCAGTTCTGTGCTGG  R: GACGGAGAGACACAAGCAGC |
| *IL-13* | F: AGCTCCCTGGTTCTCTCACT  R: CTCATTAGAAGGGGCCGTGG |
| *GAPDH* | F: AGGAGCGAGACCCCACTAACA  R: AGGGGGGCTAAGCAGTTGGT |
